# Supplementary material for: A gain of function mutation in SlNRC4a enhances basal immunity resulting in broad-spectrum disease resistance
Source: Commun Biol. 2020 Jul 30;3:404. doi: 10.1038/s42003-020-01130-w (PMC7393091; doi:10.1038/s42003-020-01130-w)
Supplement: Supplementary file 1 — Supplementary Information [file 42003_2020_1130_MOESM1_ESM.pdf]

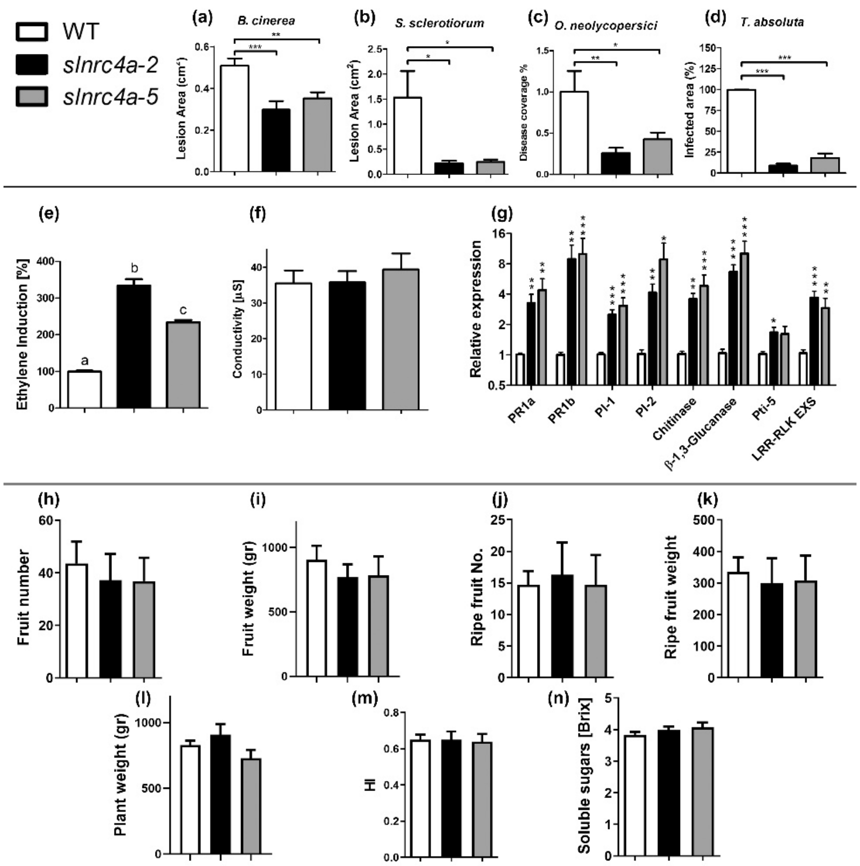

Supplementary Figure 1: Enhanced disease resistance, enhanced basal defense parameters, and agronomic traits of *slnrc4a* line#2 and *slnrc4a* line#5.

Select parameters from Figure 1- pathogen resistance (a-d), Figure 2- steady state defense parameters (e-g), and Figure 5- agricultural traits (h-n), graphed separately for two independent *slnrc4a* mutant lines: *slnrc4a-2* (black bars) and *slnrc4a-5* (gray bars).

(a-b) Lesion area was measured 3 days after inoculation with *B. cinerea* ($10X^6$ spores/mL), **(a)**, or *S. sclerotium* **(b)**. *O. neolycopersici* infection was measured as percentage of infected leaf out of total leaf area **(c)**. **(d)** Infestation was determined by counting number of insects per leaf and measuring % of infected leaf area two-weeks after *T. absoluta* exposure. **(a-d)** Average ± SEM of 3-4 independent replicates is shown. Asterisks represent statistical significance in t-test with Welch's correction (*, p-value <0.05; **, p-value <0.01; ***, p-value <0.001).
(e-g) Ethylene production of M82 and *slnrc4a* samples was measured using gas-chromatography **(e)**. M82 average ethylene production is defined as 100%. Average ± SEM of 5 independent experiments is presented. Letters represent statistical significance in t-test with Welch's correction. **(f)** Conductivity levels of M82 and *slnrc4a* samples immersed in water for 24 h was measured. Average ± SEM of 4 independent replicates is shown (one-way ANOVA, no significant difference **(g)** Gene expression analysis of pathogen responsive genes in M82 and *slnrc4a* plants was measured by RT-qPCR. Relative expression normalized to M82. Average ± SEM of three independent replicates is shown. Asterisks represent statistical significance in t-test with Welch's correction comparing each gene (*, p-value <0.05; **, p-value <0.01; ***, p-value <0.001).

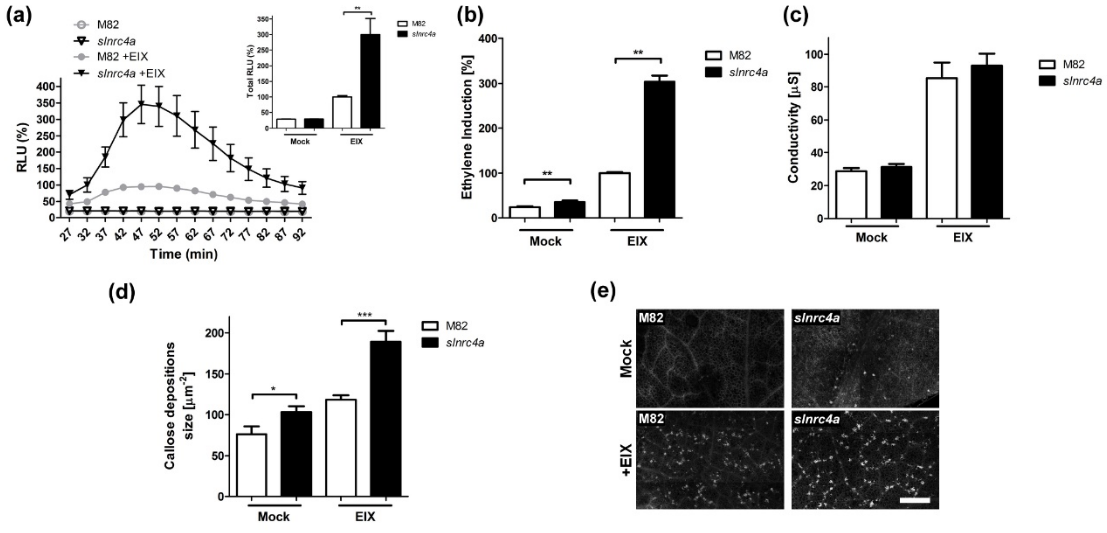

Supplementary Figure 2: Enhanced MAMP-elicited defense responses in *slnrc4a* gain of function mutants.

(a) ROS production of WT (M82) and *slnrc4a* was measured immediately after EIX application and measured every 5 min for 90 minutes using HRP-luminol method. Average ± SEM of 4 independent replicates is shown (two-way ANOVA. p-value <0.01). Inset shows total ROS production (one-way ANOVA. **, p-value <0.01).

(b) Ethylene induction after EIX elicitation in M82 and *slnrc4a* samples was measured using gas-chromatography. M82 average ethylene production after elicitation is defined as 100%, average ± SEM of 5 independent experiments is presented. Asterisks represent statistical significance in t-test with Welch's correction (**, p-value <0.01).

(c) Conductivity of M82 and *slnrc4a* samples immersed in water supplemented with EIX for 24 h was measured. Average ± SEM of 4 independent replicates is shown (one-way ANOVA. No significant difference)

(d) Twenty-four h after EIX elicitation, callose deposition was observed by confocal microscopy after aniline blue staining of M82 and *slnrc4a* leaf discs. Callose deposit size was measured using the object counting tool of ImageJ**.** Average ± SEM of three independent replicates is shown. Asterisks represent statistical significance in t-test with Welch's correction (*,p-value <0.05; ***, p-value <0.001).

(e) Callose deposition representative images. Scale bar = 200 µm.

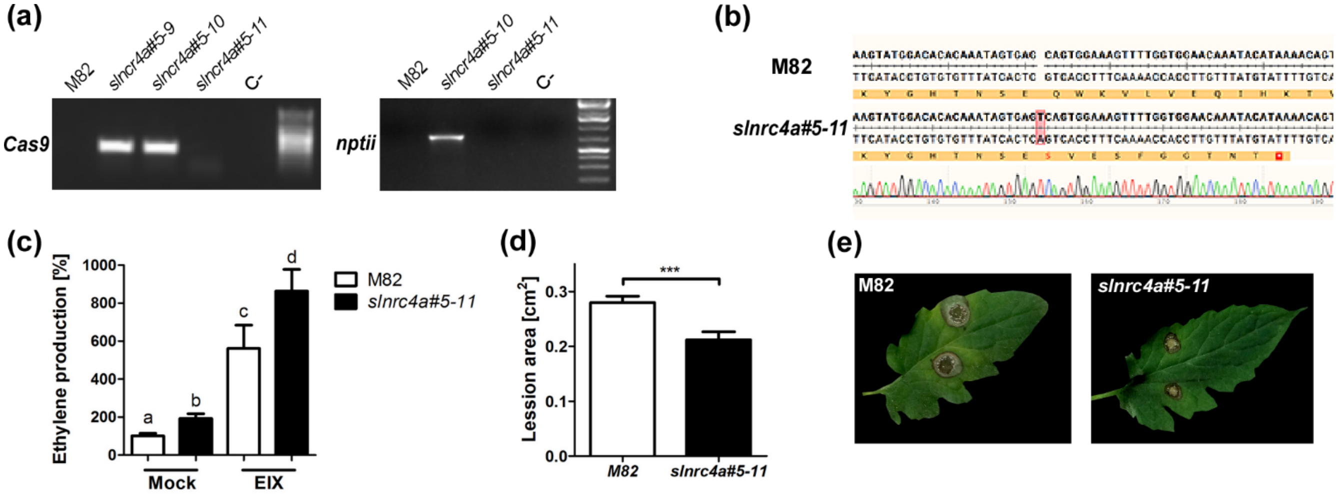

Supplementary Figure 3: Cas9-free *slnrc4a* line maintains enhanced MAMP-elicited response.

(a) PCR analysis for *Cas9* and *nptII* presence in tomatoes from the T3 generation lines of *slnrc4a-5* edited line.

(b) Alignment of slnrc4a gene sequence of M82 and slnrc4a#5-11 lines, corroborating the editing on 171 nucleotide (CDS).

(c) Ethylene induction in steady-state and after EIX elicitation in M82 and *slnrc4a* samples was measured using gas-chromatography. M82 average ethylene production is defined as 100%. Average ± SEM (N_{total}=20). Asterisks represent statistical significance in One-way ANOVA, Dunns post-test (p-value <0.05).

(d) Lesion area was measured three days after inoculation with *B. cinerea* ($10X^6$ spores/mL). Average ± SEM (N_{total}=70). Asterisk represent statistical significant in t-test with Welch's correction (***, p-value<0.001).

(e) Representative image of infection quantified in (d).

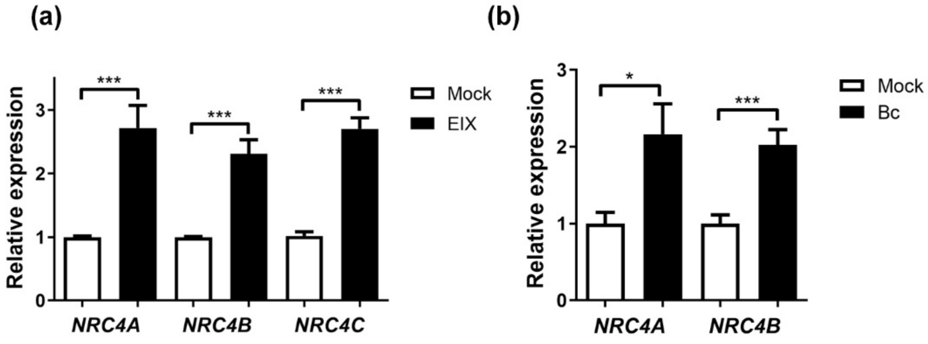

Supplementary Figure 4: Members of the NRC4 clade are induced by EIX and *B. cinerea*

Gene expression analysis of NRC4 clade members in response to EIX treatment **(a)** and *B. cinerea* inoculation **(b)** was measured in WT M82 plants by RT-qPCR. Relative expression normalized to M82 Mock. Average ± SEM of three independent replicates is shown, N>7 for each treatment. Asterisks represent statistical significance in t-test with Welch's correction comparing each gene (*, p-value <0.05; ***, p-value <0.001).

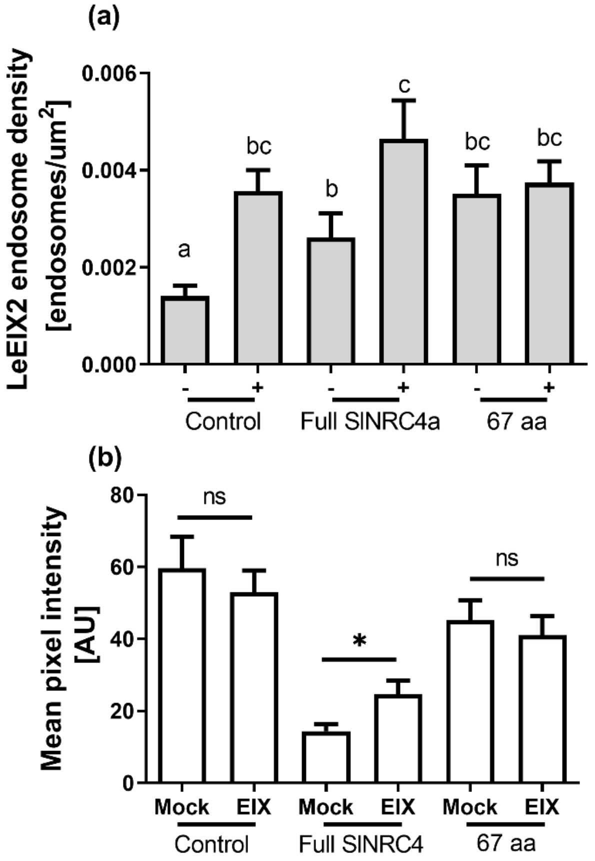

Supplementary Figure 5: The 67 aa *slnrc4a* peptide affects LeEIX2 in a ligand independent manner

N. benthamiana leaves transiently expressing LeEIX2-GFP and free mCherry (Control), the full SlNRC4a-mCherry, or the predicted 67 amino acid-mCherry (peptide present in the *slnrc4a* mutant) as indicated, were treated with EIX (1 µg g^{-1} tissue) or water (mock) at the petiole 40 hours after transformation.

(a) LeEIX2-GFP endosomes were visualized by confocal microscopy 15 minutes post EIX treatment. LeEIX2-GFP endosome density with (+) and without (-) EIX was quantified using 3D object counter (Fiji-ImageJ). Error bars represent the average ± SEM of four independent replicates, five images each. Letters indicate significant differences from the control, two-tailed t-test.

(b) mCherry, SlNRC4a-mCherry, or the predicted 67 amino acid-mCherry protein expression level (mean pixel intensity of mCherry signal) was quantified using FIJI-ImageJ. Sixteen images from four experiments were analyzed. Error bars represent the average ± SEM. Asterisk indicates significant difference (two-tailed t-test, $P < 0.05$).

Data adapted from Leibman-Markus et al., 2018b.

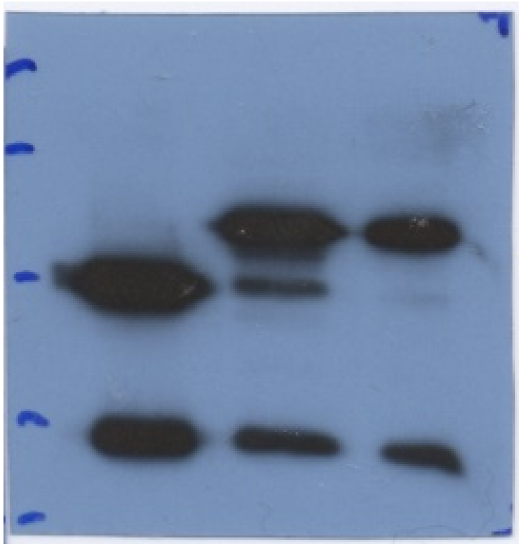

Supplementary Figure 6: Original uncropped image of the blot presented in Figure 7c.

Supplementary Table 1: Analysis of volatile metabolites in M82 (WT) and *slnrc4a* lines. Concentration of volatile compounds in M82 (WT) and *slnrc4a* plants were quantified using Gas Chromatography (ng per g of fresh weight = ng/gfw). The values are also expressed percentage of the concentration detected in WT. Asterisks represent statistical significance in t-test with Welch's correction (***, p-value <0.001; **, p-value <0.01; *, p-value <0.05).

GROUP	Compound	WT			*slnrc4a*			T-test Welch's correction	
		Average [ng/gfw]	SD	%	Average	SD	%	p-Value	
Fatty acid	2,3- Butanediol	11.6	7.3	100	100.3	48.5	868.2	0.0003	***
	Penten-3-ol	42.6	17.8	100	65.2	8.0	153.0	0.0963	NS
	1-Penten-3-one	35.0	6.5	100	61.1	24.7	174.6	0.0125	*
	Pentanal	48.8	15.3	100	35.0	29.3	71.7	0.3321	NS
	Hexanal	442.7	111.4	100	299.6	149.6	67.7	0.127	NS
	2E-Hexenal	2149.8	370.8	100	2113.1	448.9	98.3	0.8989	NS
	Hexanol	245.4	51.2	100	136.1	27.2	55.4	0.0137	*
	2E- Heptenal	153.6	49.0	100	160.4	21.3	104.4	0.8418	NS
	2E,4E- Heptadienal	118.3	36.5	100	74.8	22.0	63.3	0.1161	NS
Monoterpene	alpha-Pinene	669.9	280.3	100	1146.2	199.6	171.1	0.0372	*
	3,7,7-trimethyl-1,3,5-Cycloheptatriene	417.6	115.0	100	684.8	80.0	164.0	0.0086	**
	b-Pinene	1238.3	208.7	100	1171.6	113.2	94.6	0.651	NS
	d- 2-Carene	1581.0	371.2	100	2511.4	245.1	158.9	0.0055	**
	α-Phellandrene	685.3	151.1	100	1036.4	105.6	151.2	0.0086	**
	α-Terpinene	131.6	25.2	100	179.5	21.4	136.4	0.0235	*
	p-Cymene	190.6	23.2	100	269.6	32.2	141.4	0.001	**
	b- Phellandrene	5879.7	1036.4	100	8824.2	847.5	150.1	0.003	**
	γ-Terpinene	60.8	7.6	100	116.6	19.5	191.8	< 0.0001	***
	Terpinolene	23.8	4.6	100	34.0	4.1	142.4	0.012	*
	Cryptone	46.2	11.4	100	75.1	14.1	162.5	0.0069	**
sesquiterpene	E-Caryophyllene	119.1	21.4	100	196.3	54.7	164.8	0.0042	**
	Humulen-(v1)	39.5	9.6	100	66.1	11.3	167.2	0.0038	**
	α-Humulene	86.6	8.3	100	115.7	28.0	133.6	0.0163	*
phenylpropanoid	Benzaldehyde	429.5	30.7	100	249.0	59.6	58.0	< 0.0001	***
	Benzyl alcohol	93.4	32.7	100	113.1	27.3	121.1	0.4169	NS
	o- Guaiacol	190.2	82.2	100	212.6	73.9	111.8	0.7122	NS
	Phenyl ethyl alcohol	384.9	31.2	100	572.3	93.9	148.7	0.0002	***
	Methyl salicylate	743.4	163.4	100	1004.0	113.3	135.1	0.0471	*
	Eugenol	40.0	9.7	100	64.3	15.8	160.7	0.0102	*
carotenoid derivatives	b-Cyclocitral	211.8	46.1	100	305.7	33.4	144.4	0.0169	*
	b- Ionone	481.1	128.1	100	627.8	116.1	130.5	0.1418	NS

Supplementary Table 2: Primers used in this work.

Locus	Name	Forward	Reverse	Reference
Solyc01g106620	PR1a	CTGGTGCTGTGAAGATGTGG	TGACCCTAGCACAACCAAGA	(Harel et al., 2014)
Solyc00g174340	PR1b	GTGTCCGAGAGGCCAAGCTA	AGGACGTTGTCCGATCCAGTT	
Solyc01g097270	Pathogen induced 1	TGCTTAAGGGTGACAAATACA CG	ACATTCACATTGTCACCGCA	
Solyc03g020050	Proteinase inhibitor 2	CGACGTGTTGCACTGGTTAC	TGCCAATCCAGAAGATGGAC	(Harel et al., 2014)
Solyc10g055800	Chitinase 2	AATGGTGGCCTAGAACGTGG	AGCTGAGTCCAACAGACTACA	
Solyc01g060020	beta-1,3-glucanase	TCGAACAGGAGGAGGATCTG	TCCAGGCTTTCTCGGACTAC	(Harel et al., 2014)
Solyc02g077370	Pti 5	GACATGGTGCGAGAGTATGG	CTGAAACAGAGGCGTTCACT	(Harel et al., 2014)
Solyc07g008600	LRR-RLK-EXS	TCAGTAGGGCTCGCTAACCT	GAAGAGGAGGGCCACATAGC	
Solyc03G123860	RLK-INRPK1c	TGCTACTCTAGGCCAGCTCA	TGCAACTGGGTGAGTGATCC	(Yang et al., 2017)
Solyc02G070890	FLS2	GGGTTGGGGCAGTTATCCAA	GGTGGAATGGCACCTGAGAA	
Solyc08G016310	LRR-RLP	TCACTGGGGAGATTCCGAGA	GTTCCAGTCCACCACCCAAT	(Yang et al., 2017)
