# Supplementary material for: A gain of function mutation in SlNRC4a enhances basal immunity resulting in broad-spectrum disease resistance
Source: Commun Biol. 2020 Jul 30;3:404. doi: 10.1038/s42003-020-01130-w (PMC7393091; doi:10.1038/s42003-020-01130-w)
Supplement: Supplementary file 2 — Reporting Summary [file 42003_2020_1130_MOESM2_ESM.pdf]

nature research

Corresponding author(s): Maya Bar

Last updated by author(s): Jul 1, 2020

Reporting Summary

Nature Research wishes to improve the reproducibility of the work that we publish. This form provides structure for consistency and transparency in reporting. For further information on Nature Research policies, see Authors & Referees and the Editorial Policy Checklist.

Statistics

For all statistical analyses, confirm that the following items are present in the figure legend, table legend, main text, or Methods section.

n/a	Confirmed	
☐	☒	The exact sample size (*n*) for each experimental group/condition, given as a discrete number and unit of measurement
☐	☒	A statement on whether measurements were taken from distinct samples or whether the same sample was measured repeatedly
☐	☒	The statistical test(s) used AND whether they are one- or two-sided<br>*Only common tests should be described solely by name; describe more complex techniques in the Methods section.*
☐	☒	A description of all covariates tested
☐	☒	A description of any assumptions or corrections, such as tests of normality and adjustment for multiple comparisons
☐	☒	A full description of the statistical parameters including central tendency (e.g. means) or other basic estimates (e.g. regression coefficient) AND variation (e.g. standard deviation) or associated estimates of uncertainty (e.g. confidence intervals)
☒	☐	For null hypothesis testing, the test statistic (e.g. *F*, *t*, *r*) with confidence intervals, effect sizes, degrees of freedom and *P* value noted<br>*Give P values as exact values whenever suitable.*
☒	☐	For Bayesian analysis, information on the choice of priors and Markov chain Monte Carlo settings
☒	☐	For hierarchical and complex designs, identification of the appropriate level for tests and full reporting of outcomes
☒	☐	Estimates of effect sizes (e.g. Cohen's *d*, Pearson's *r*), indicating how they were calculated

Our web collection on statistics for biologists contains articles on many of the points above.

Software and code

Policy information about availability of computer code

Data collection	*Provide a description of all commercial, open source and custom code used to collect the data in this study, specifying the version used OR state that no software was used.*
Data analysis	*Provide a description of all commercial, open source and custom code used to analyse the data in this study, specifying the version used OR state that no software was used.*

For manuscripts utilizing custom algorithms or software that are central to the research but not yet described in published literature, software must be made available to editors/reviewers. We strongly encourage code deposition in a community repository (e.g. GitHub). See the Nature Research guidelines for submitting code & software for further information.

Data

Policy information about availability of data

All manuscripts must include a data availability statement. This statement should provide the following information, where applicable:

- Accession codes, unique identifiers, or web links for publicly available datasets
- A list of figures that have associated raw data
- A description of any restrictions on data availability

The authors declare that the data supporting the findings of this study are available within the paper and its supplementary information files. Raw data is available from the corresponding author upon reasonable request.

Field-specific reporting

Please select the one below that is the best fit for your research. If you are not sure, read the appropriate sections before making your selection.

☒ Life sciences ☐ Behavioural & social sciences ☐ Ecological, evolutionary & environmental sciences

For a reference copy of the document with all sections, see nature.com/documents/nr-reporting-summary-flat.pdf

Life sciences study design

All studies must disclose on these points even when the disclosure is negative.

Sample size	A minimum of 3 biological repeats, each including a minimum of 3 technical repeats, was employed- as is standard practice in the life sciences. Each experiment was repeated at least 3 times. In all experiments, N>18 for all treatments except in the metabolomic analyses, where two technical replicates were used per experiment (N=6), however, in this case, each replicate was composed of 4 biological replicates pooled together, such that 12 plants in total were sampled per genotype.
Data exclusions	No data was excluded.
Replication	A minimum of 3 biological repeats, each including a minimum of 3 technical repeats, was employed- as is standard practice in the life sciences. Each experiment was repeated at least 3 times.
Randomization	No group allocation was required, however, samples were analyzed in randomized order where applicable and not as "blocks" of samples.
Blinding	Blinding during data collection is not always possible, however, analyses were conducted blindly by labeling treatments with random numbers.

Reporting for specific materials, systems and methods

We require information from authors about some types of materials, experimental systems and methods used in many studies. Here, indicate whether each material, system or method listed is relevant to your study. If you are not sure if a list item applies to your research, read the appropriate section before selecting a response.

Materials & experimental systems

n/a	Involved in the study	
☐	☒	Antibodies
☒	☐	Eukaryotic cell lines
☒	☐	Palaeontology
☒	☐	Animals and other organisms
☒	☐	Human research participants
☒	☐	Clinical data

Methods

n/a	Involved in the study	
☒	☐	ChIP-seq
☒	☐	Flow cytometry
☒	☐	MRI-based neuroimaging

Antibodies

Antibodies used	Mouse anti-RFP RFP-antibody 6G6 (used for monomeric cherry detection), Chromotek, Planegg-Martinsried, Germany
Validation	RFP-antibody 6G6: Mouse monoclonal 6G6 to RFP, tested by the manufacturer on mRFP, mCherry, mPlum, mOrange, mRFPruby, dsRed, mScarlet, tdTomato, for western blotting at 1:1000 ratio- validated on an extract of HEK cells transiently expressing the different listed fluorescent proteins.

October 2018
